# Endovascular Repair for a Ruptured AAA due to a Combined Type IIIb and Ia Endoleak

**DOI:** 10.1155/2018/1502328

**Published:** 2018-04-24

**Authors:** Konstantinos Ioannis Avgerinos, Nikolaos Melas, Athanasios Saratzis, Marianthi V. Tympanidou, Nikolaos Saratzis, Ioannis Lazaridis

**Affiliations:** ^1^Department of Medicine, Faculty of Health Sciences, Aristotle University of Thessaloniki, Thessaloniki, Greece; ^2^1st Department of Surgery, Aristotle University of Thessaloniki, Papageorgiou General Hospital, Thessaloniki, Greece

## Abstract

We report a case of a ruptured abdominal aortic aneurysm (AAA) caused by a combined type IIIb and Ia endoleak. Also, we propose the mechanism that resulted in this combined endoleak. Specifically, a 71-year old-man, with a previous history of endovascular aneurysm repair (EVAR) for an AAA, was diagnosed with a contained rupture. CT scan depicted a type Ia endoleak and a migrated Talent endograft. A proximal aortic cuff sealed the endoleak, but intraoperative angiography revealed that a type IIIb endoleak coexisted due to fabric tear close to the Talent bifurcation. A second aortic cuff could not seal the fabric tear; so, in-lay parallel limbs were sequentially deployed as a “kissing endograft” technique inside the cuff. Simultaneous treatment of combined type IIIb and Ia endoleaks has not yet been described. Maybe the type IIIb endoleak is the primary entity causing sac enlargement, neck recontouring, proximal migration, and ultimately type Ia endoleak, which leads to huge enlargement and rupture. Placement of an aortic cuff to seal the proximal endoleak/migration and kissing endografts limbs for the fabric tear seems a safe option in such patients.

## 1. Introduction

Endoleak is considered the Achilles heel of endovascular aortic repair (EVAR). Endoleak classification has been described elsewhere in detail [1]. Type I (a and b) is associated with a poor sealing zone and can arise immediately after the initial implantation or during the late follow-up [2]. Type III endoleak could be due to limb dislocation (IIIa) or as a consequence of fabric tear (type IIIb). The latter type has been clearly described with various types of endografts as a late complication [3, 4]. Both type I and III endoleaks usually lead to sac enlargement and, if left untreated, to aneurysmal rupture and death. In most instances, endoleaks are of one isolated type. In this article, we present a case of a ruptured AAA due to a combination of type IIIb and Ia endoleaks. We also propose the potential mechanism that type IIIb endoleak can lead to sac enlargement, proximal neck recontouring, proximal migration, type Ia endoleak, rapid sac enlargement, and ultimately rupture. Moreover, we describe the potential difficulties to treat such combined endoleaks and we propose an easily accessible configuration from within commercially available endografts for such cases.

## 2. Case Presentation

**Figure 1:**
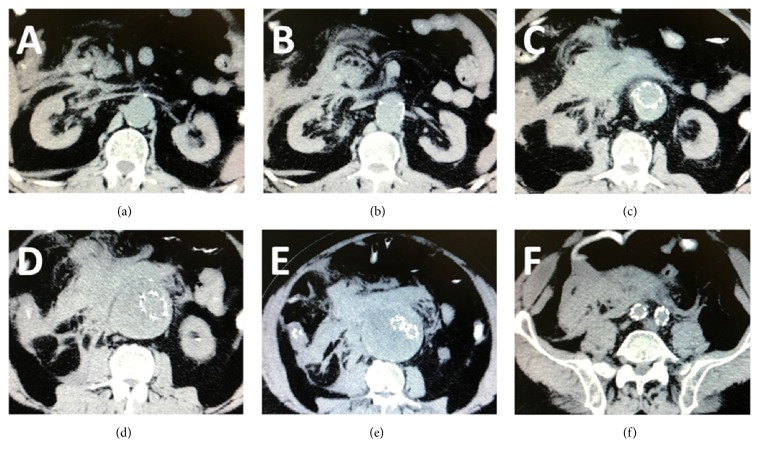
Preoperative CT scan. Contrast media had to be avoided due to severe renal failure. The Talent stent-graft was migrated and the proximal sealing was lost ((a)–(c)) leading to potential type I endoleak, sac enlargement, and rupture. A leaking 9 cm AAA with a huge retroperitoneal hematoma was depicted ((d) and (e)). Distal iliac sealing was sufficient (f).

**Figure 2:**
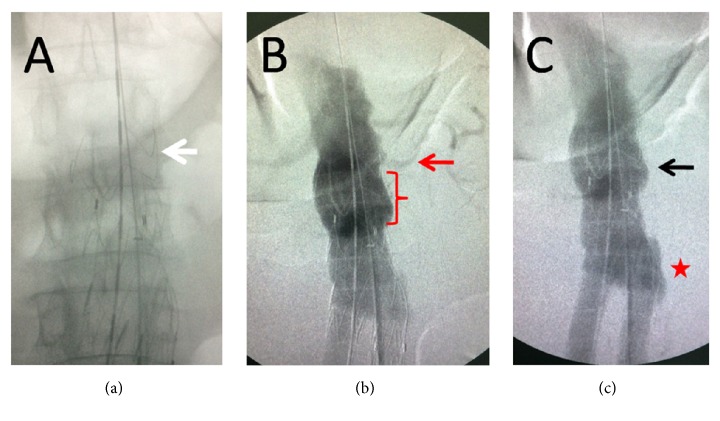
Initial intraoperative angiography. The Talent suprarenal crown was found to be damaged ((a), white arrow), the endograft was migrated 2 cm distally to the left renal artery ((b), red arrow and red bracket), and the proximal sealing was lost and a type Ia endoleak was shown ((c) black arrow). At the moment the second endoleak ((c), red star) pool was advocated as a continuation from the Ia endoleak.

**Figure 3:**
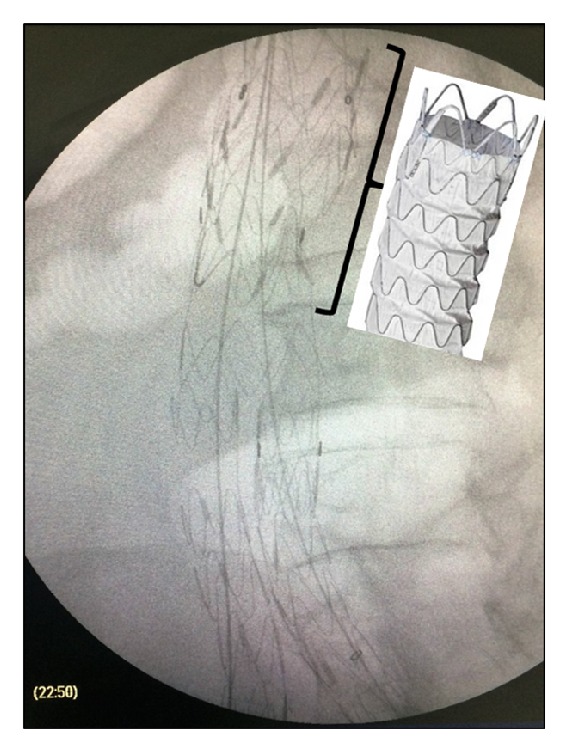
A 28 × 60 Ankura aortic extender was deployed to bridge the proximal remaining aortic neck with the migrated Talent body.

**Figure 4:**
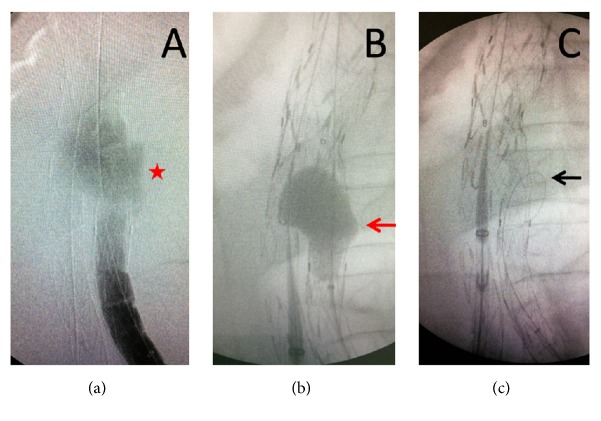
Contrast media continued to be visible outside the Talent endograft after the Ankura cuff deployment, restricted at the level of its bifurcation ((a), red star). Balloon inflation did not seal the endoleak; on the contrary, a fabric tear becomes apparent near the stent-graft bifurcation feeding the endoleak ((b), red arrow). A guide wire was passed through the fabric opening for verification ((c), black arrow).

**Figure 5:**
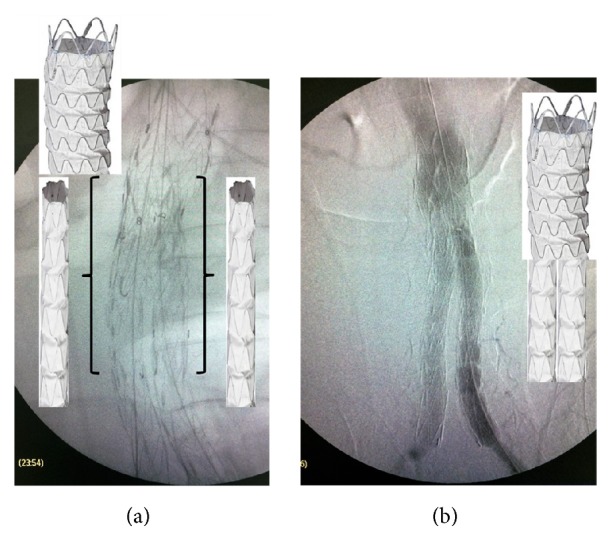
(a) Two Ankura limbs, 16-16 × 100, were simultaneously deployed as parallel kissing endografts. The proximal 6 cm of the parallel endografts was positioned above the Talent bifurcation within the Ankura cuff and the distal 4 cm inside each Talent limb. (b) Completion of angiogram verified endoleak resolution, renal and iliac artery patency, and proper endograft deployment.

A 71-year-old male, who had been submitted to EVAR with a bifurcated Talent endoprosthesis in 2006 for a 5,5 cm AAA, was admitted to the emergency department reporting sudden onset of abdominal pain with lumbar radiation and a history of a brief loss of consciousness one hour earlier. He was found to be hypotensive and tachycardic (BP: 90/60 mmHg, Bpm: 110/min, RR: 22/min, and SpO2: 95%). Hgb level was significantly decreased (8,5 g/dl) and a pulsatile mass was prominent upon clinical examination. The patient's medical history included chronic obstructive pulmonary disease, myocardial infarction with heart failure, chronic renal failure, hypertension, and smoking. The high clinical suspicion of ruptured AAA was verified with CT scan. Contrast media had to be avoided due to severe renal failure. A leaking 9 cm AAA with a huge retroperitoneal hematoma was depicted (Figure 1). The Talent stent-graft was migrated (Figure 1) and the proximal sealing was lost, leading to potential type I endoleak, sac enlargement, and rupture. At this point, no further information was available from the CT scan (unenhanced). Endoluminal reintervention was immediately scheduled with the use of a proximal aortic extension. The procedure was carried out in an operating room equipped with a portable C-arm (Siremobil 2000, Siemens, Erlangen, Germany) under local anesthesia. Bilateral femoral arteries were surgically exposed in standard fashion. Heparin (5000 IU) was administered intravenously. Initial angiography verified the hypothesis of migration and endoleak. The Talent was migrated 2 cm distally to the left (lower) renal artery, the proximal sealing was lost, and a huge type Ia endoleak was shown (Figure 2). Based upon the CT measurements and the intraoperative data, a 28 mm in diameter × 60 mm in length Ankura (LifeTech, Inc., China) aortic extender with 15 mm bare stent was deployed (Figure 3) to bridge the proximal aortic neck (estimated 24 mm in diameter × 15 mm in length) with the migrated Talent body (26 mm in diameter × 50 mm in length tubular segment). Postdeployment angiography showed that the type Ia endoleak was sealed and the aortic extender was correctly placed just below the left renal artery. To our surprise, contrast media continued to be visible in a different fashion from the initial angiography outside the Talent endograft restricted at the level of its bifurcation (Figure 4). When we inflated a molding balloon thinking that it was a leak in between the Ankura extender and the Talent body, a fabric tear became apparent near the stent-graft bifurcation (Figure 4) feeding the endoleak. The balloon was removed and a guide wire was meticulously passed through the fabric opening to verify the fabric tear and the type IIIb endoleak (Figure 4). Two Ankura limbs, 16-16 × 100, were inserted and simultaneously deployed as parallel kissing endografts. The proximal 6 cm of the parallel endografts was positioned above the Talent bifurcation within the Ankura cuff and the distal 4 cm inside each Talent limb below the Talent bifurcation (Figure 5) to ensure adequate sealing of the fabric tear. Kissing balloon technique followed using two 14 × 40 PTA Atlas balloons (Bard Peripheral Vascular Inc., AZ, USA) inflated at 4 atm. Completion of DSA verified endoleak resolution, renal and iliac artery patency, and proper endograft deployment. The patient recovered fully and was released on the 7th postoperative day with no further renal deterioration and increased Hgb level (11,5 g/dl). The patient was readmitted in another hospital in the 40th postoperative day due to pulmonary edema. Ultimately he died due to heart failure and his death had no relation to the AAA. The report of this man's case was approved by the institutional review board.

## 3. Discussion

Endovascular aortic repair is associated with less perioperative mortality than open aortic surgery, particularly in aged patients. However, EVAR has a higher long-term complication rate. Endoleak is a potential complication of EVAR which could jeopardize surgical success of EVAR [1]. It can happen because of leakage at sealing zones (types Ia, Ib, and Ic), leakage due to side branches arising from within the aneurismal sac (types IIa and IIb), leakage from graft defects (types IIIa and IIIb), and leakage due to graft porosity (type IV) [1]. Additionally, we can have aneurysm sac enlargement (endotension) without detectable endoleak (type V) [1]. Endoleaks are also categorized to early or late type, depending on the time of occurrence [1]. Some types of endoleak are directly connected to serious complications, aneurysm 97 rupture, and even death [5]. Specifically, types I and III require immediate intervention with endovascular techniques and, if needed, conversion to open surgery [6]. For types II, IV, and V, which are low pressure endoleaks, urgent treatment is not mandatory [7]. Regarding type III endoleak, there are two subtypes, IIIa and IIIb, as it is already mentioned. The former is due to junctional leak of the graft (limb dislocation), while the latter is due to fabric tear [1]. Generally, type III endoleaks occur rarely [8]. It is known that various devices have been approved for EVAR by FDA. Already, seven cases of type IIIb endoleak after placement of Zenith endografts have been reported in literature. Of these, two were early and five were late endoleaks. The first case of IIIb endoleak with a Zenith endograft was reported by Wanhainen et al. [3]. For Ancure endograft, three cases of late type IIIb endoleak have been reported in two studies. The two cases were reported by Teutelink et al. [9]. For Endurant, Excluder, and AneuRx grafts, one case of IIIb endoleak for each of them has been described. Endoleak was of early type for the Endurant graft and of late type for the Excluder and AneuRx grafts [4, 10, 11]. For Talent endograft, two cases of IIIb endoleak, one early and one late, have been described [12, 13]. The case of the early Talent endoleak occurred intraoperatively in a 62-year-old patient and was repaired with a Zenith endograft placement [12]. Twelve months after the procedure, the patient did not have any symptoms but the CT scan revealed mild proximal kinking of the upper end of the device. The late Talent endoleak case occurred in a 83-year-old man seven years after the primary procedure and was successfully treated with an Endurant endograft [13]. Two days later, the patient was discharged. Finally, Duvnjak reported a case where there was a combination of type Ia and IIIb endoleaks two years after the placement of a Talent endograft in a 75-year-old man [14]. Initially, only the type Ia endoleak was managed, as the type IIIb endoleak was not easily apparent and it was underestimated in the first place. For the first endoleak, a fenestrated custom-made aortic cuff (Cook, Bloomington, IN) was used, whereas for the latter type, one month later, an aortouniiliac converter device (Cook) was used, together with a femorofemoral bypass for perfusion of the opposite limb [14]. The unique feature of this case report is the coexistence of combined type Ia and IIIb endoleaks and the simultaneous treatment of both entities during the same session. It is a word of caution that the IIIb endoleak may be obscured or underestimated in the preoperative imaging and even during the intraoperative angiogram, like in Duvnjak case or in our case as well [14]. Moreover, the case shows an “off the shelves” solution to treat emergency situation like AAA rupture for similar cases, since special bifurcated endografts are lacking to be used as an “inlay” technique. If an additional cuff was deployed, distally to the initial one, it would not solve the type IIIb endoleak, since the tear was very close to the bottom of the tubular segment almost at the beginning of the left limb (Figure 4), so it was rejected. A bifurcated endograft could not be used as well, since the contralateral stump would not be able to open correctly or might be compressed due to the short distance between lower renal and Talent graft bifurcations. All the bifurcated endografts offer 4 or 5 cm main body plus 3 cm contralateral stump. So, the only possible bail-out solution was to deploy two parallel endograft limbs as a kissing stent-graft technique. Appropriate oversizing (2 × 16 mm in the 28 cuffs) and adequate length (100 mm) of the limbs avoided gutter formation and ultimately sealed the fabric tear. Here, we would like to propose the mechanism that may cause such combined endoleaks. The possible mechanism is briefly described in the next scheme: fabric and frame fatigue → fabric tear → type IIIb endoleak → sac enlargement → neck recontouring → graft migration → proximal type Ia endoleak → greater sac enlargement → rupture. Although such cases have not been reported in the past, they might have been existed, but the most obvious type Ia endoleak might obscured the type IIIb endoleak, something that initially misled us as well. Combined endoleaks constitute a separate entity and should be approached in a different fashion, because sealing only one of them might not solve the whole problem after all as what happened in Duvnjak case [14]. In our case, if the fabric tear was not situated near the bifurcation but 2 cm proximally, the Ankura cuff would seal both endoleaks and the type IIIb endoleak would go undiagnosed. Meticulous interpretation of intraoperative angiograms is important to solve such difficult and rare cases. Awareness and anticipation are mandatory especially in endoleaks over the long term where material fatigue is more frequent. Finally, strict follow-up of patients after EVAR should not be avoided.
